# Responses of leaf gas exchange attributes, photosynthetic pigments and antioxidant enzymes in NaCl-stressed cotton (*Gossypium hirsutum* L.) seedlings to exogenous glycine betaine and salicylic acid

**DOI:** 10.1186/s12870-020-02624-9

**Published:** 2020-09-21

**Authors:** Abdoul Kader Mounkaila Hamani, Guangshuai Wang, Mukesh Kumar Soothar, Xiaojun Shen, Yang Gao, Rangjian Qiu, Faisal Mehmood

**Affiliations:** 1 Farmland Irrigation Research Institute, Chinese Academy of Agriculture Sciences/Key Laboratory of Crop Water Use and Regulation, Ministry of Agriculture and Rural Affairs, Xinxiang, Henan 453002 PR China; 2 Graduate School of Chinese Academy of Agricultural Sciences, Beijing, 100081 PR China; 3 Collaborative Innovation Center on Forecast and Evaluation of Meteorological Disasters, Jiangsu Key Laboratory of Agricultural Meteorology, Nanjing University of Information Science and Technology, Nanjing, 210044 China

**Keywords:** Antioxidant, Cotton, Gas exchange, Salt stress, Glycine betaine, Salicylic acid

## Abstract

**Background:**

Application of exogenous glycine betaine (GB) and exogenous salicylic acid (SA) mitigates the adverse effects of salinity. Foliar spraying with exogenous GB or SA alleviates salt stress in plants by increasing leaf gas exchange and stimulating antioxidant enzyme activity. The effects of foliar application of exogenous GB and SA on the physiology and biochemistry of cotton seedlings subjected to salt stress remain unclear.

**Results:**

Results showed that salt stress of 150 mM NaCl significantly reduced leaf gas exchange and chlorophyll fluorescence and decreased photosynthetic pigment quantities and leaf relative water content. Foliar spray concentrations of 5.0 mM exogenous GB and 1.0 mM exogenous SA promoted gas exchange and fluorescence in cotton seedlings, increased quantities of chlorophyll pigments, and stimulated the antioxidant enzyme activity. The foliar spray also increased leaf relative water content and endogenous GB and SA content in comparison with the salt-stressed only control. Despite the salt-induced increase in antioxidant enzyme content, exogenous GB and SA in experimental concentrations significantly increased the activity of glutathione reductase, ascorbate peroxidase, superoxide dismutase, catalase and peroxidase, and decreased malondialdehyde content under salt stress. Across all experimental foliar spray GB and SA concentrations, the photochemical efficiency of photosystem II (*F*_*V*_/*F*_*M*_) reached a peak at a concentration of 5.0 mM GB. The net photosynthetic rate (*P*_*n*_) and *F*_*V*_/*F*_*M*_ were positively correlated with chlorophyll a and chlorophyll b content in response to foliar spraying of exogenous GB and SA under salt stress.

**Conclusions:**

We concluded, from our results, that concentrations of 5.0 mM GB or 1.0 mM SA are optimal choices for mitigating NaCl-induced damage in cotton seedlings because they promote leaf photosynthesis, increase quantities of photosynthetic pigments, and stimulate antioxidant enzyme activity. Among, 5.0 mM GB and 1.0 mM SA, the best performance in enhancing endogenous GB and SA concentrations was obtained with the foliar application of 1.0 mM SA under salt stress.

## Background

The phenomenon of soil salinization is intensifying worldwide due to the extensive irrigation practices with saline water, ongoing sea-level rise, and large-scale soil erosion [1]. An elevated concentration of salt in soil (due mainly to NaCl) represents the most intense form of abiotic stress; it restricts plant productivity on about 20% of irrigated lands worldwide [2, 3]. Glycine betaine (GB) is an osmolyte that plays a crucial role in plant response to various abiotic stresses, including cold stress and high salinity [4]. Salicylic acid (SA) is an essential phytohormone that adjusts several physiological and biochemical plant processes in saline conditions. SA has various effects on abiotic stress tolerance mechanisms that respond to stressors such as heat, heavy metals, ozone, and osmotic stress [5].

Cotton is the world’s most important textile fiber, and the cotton plant is extremely resilient to salt stress. However, cotton plants grown in saline conditions show several negative effects on their growth, such as reduced fiber quality and yield. The effects of salinity occur mainly at germination and during the seedling stage [6, 7]. To reduce these effects, an understanding of the cotton plant responses to salt stress is necessary. GB and SA alleviate salt stress, and it is desirable to investigate how foliar applications of exogenous GB and SA affect the resistance of cotton seedlings to salt stress.

Leaf photosynthesis is most critically affected by salt stress. Salt-induced reduction in leaf photosynthesis is due to stomatal closure as well as decreased concentration of intracellular CO_2_ and some other non-stomatal characteristics. The stomatal closure restricts CO_2_ availability in the leaves and transpiration rate (*T*_*r*_). As a result, the Intercellular CO_2_ concentration (*C*_*i*_) declines and causes altered leaf biochemistry which negatively affects the net photosynthetic rate (*P*_*n*_) under prolonged salt stress conditions. Moreover, the stomatal conductance (*g*_*s*_) and *P*_*n*_ concurrently decrease under various saline conditions [8–10]. There is evidence that salt stress influences key photosynthetic enzyme activities as well as chlorophyll and carotenoid content [11]. Salt stress reduces the efficiency of photosynthesis in a mechanism that seems to be related to the photosystem II complex and is sensitive to all types of stresses [12]. Quantification of chlorophyll fluorescence is reported to be an accurate method of identifying and the environmental stress tolerance of a plant [13, 14]. Foliar application of exogenous GB to plants that are unable to synthesize GB or are able to synthesize only small quantities of GB can alleviate the impacts of abiotic stress [15]. In one study, exogenous GB was applied to corn leaves to mitigate the harmful effects of salinity, whether able to synthesize GB or not [16]. Foliar application of exogenous SA promoted many physiological responses in plants grown in saline conditions, including increases in *P*_*n*_, *g*_*s*_, *T*_*r*_, and quantum efficiency of photochemical transports used for photosynthesis (Φ_*PSII*_) [5, 17]. Several studies have reported that exogenous SA was a critical plant growth regulator and increased plant resilience to salt stress [18, 19].

Antioxidant enzymes such as superoxide dismutase (SOD), catalase (CAT), and peroxidase (POD) increase plant resistance to salt stress by mitigating the effects of oxidative injuries [20]. Plants possess various antioxidant enzymes that protect plant cells from potential cytotoxins. The relationship between salt resistance and antioxidant enzyme activity has been experimentally investigated in leaves of 8 weeks old cotton plants of both saline resistant (*Gossypium hirsutum* L. cv. Acala 1517-8-8) and saline sensitive (*G. hirsutum* cv. Deltapine 50) varieties [21]. Previous studies have investigated the effects of high NaCl concentration (150 mM) on hypocotyl-derived callus of *G. hirsutum* cv. Acala 1517-8-8 and found variations in antioxidant enzymes. The activity of the enzymes SOD, CAT, ascorbate peroxidase (APX), and glutathione reductase (GR) increased [21]. Malondialdehyde (MDA) accumulation is another important indicator of oxidative stress-induced lipid peroxidation in plants subjected to stress [22]. Previous investigations found that exogenous GB sprayed on plants alleviated the adverse effects of salt stress on tobacco (*Nicotiana tabacum* L. cv. Bright Yellow 2) suspension-cultured cells by sustaining or increasing the activities of antioxidants in response to oxidative stress caused by salt [23]. Exogenous SA sprayed on plants was found to control the activities of antioxidant enzymes and improve plant resistance to abiotic stress [24].

We found that no comprehensive studies have been conducted to assess the physiological and biochemical responses of cotton seedlings to exogenous GB and SA application in saline conditions. We hypothesized that increased cotton seedling leaf gas exchange in a 150 mM NaCl regime due to foliar application of exogenous GB and SA would increase antioxidant enzyme activity and thus increase the resistance of cotton seedlings to salt stress. Accordingly, the main goal of this study was to assess the effects of foliar application of exogenous GB and SA on leaf gas exchange, chlorophyll fluorescence, photosynthetic pigment content and antioxidant enzymes in a cotton genotype grown in a 150 mM NaCl concentration salt stress regime.

## Results

### Responses of endogenous GB and SA to exogenous GB and SA

Endogenous GB and SA content were measured in cotton leaves after they were harvested. The application of 150 mM NaCl (treatment ST; see Table 1) significantly increased both endogenous GB and SA concentrations by 51 and 90%, in comparison with the control treatment (CK). The foliar applications of all concentrations of exogenous GB and SA, except for the 1.0 mM SA (treatment SA1.0), significantly increased endogenous GB content when compared with the NaCl-stressed only treatment (ST). Endogenous SA content significantly increased under salt stress in foliar spraying treatments SA1.5 and SA2.0, but decreased in all foliar spraying GB treatments (GB2.5, GB5.0 and GB7.5) in comparison with the saline only treatment (ST); see Table 1. The highest accumulation of endogenous GB in cotton seedling leaves was given by treatment GB7.5. The highest accumulation of endogenous SA was given by treatment SA2.0.

**Table 1:** Endogenous glycine betaine (GB) and salicylic acid (SA) content for exogenous GB and SA treatments under a 150 mM NaCl concentration regime

Treatment label	GB content (mg g^−1^FW)	SA content (μg g^−1^FW)
CK	14.12 ± 1.95 f	1.01 ± 0.002 d
GB2.5	31.63 ± 1.67 de	0.41 ± 0.001 d
GB5.0	35.38 ± 1.23 bc	0.64 ± 0.001 d
GB7.5	42.27 ± 1.25 a	0.43 ± 0.001 d
SA1.0	38.52 ± 1.90 b	9.98 ± 1.654 c
SA1.5	34.61 ± 1.20 cd	21.51 ± 1.033 b
SA2.0	28.44 ± 1.67 e	27.87 ± 1.973 a
ST	28.45 ± 1.71 e	9.95 ± 1.048 c

*FW* Fresh weight, *CK* Control, *GB2.5* 2.5 mM GB, *GB5.0* 5.0 mM GB, *GB7.5* 7.5 mM GB, *SA1.0* 1.0 mM SA, *SA1.5* 1.5 mM SA, *SA2.0* 2.0 mM SA, *ST* saline treatment (150 mM NaCl)

All data are mean ± standard deviation. Differences between treatments indicated by different letters in each column are significant at *P* < 0.05

### Response of leaf gas exchange to exogenous GB and SA

**Fig. 1:**
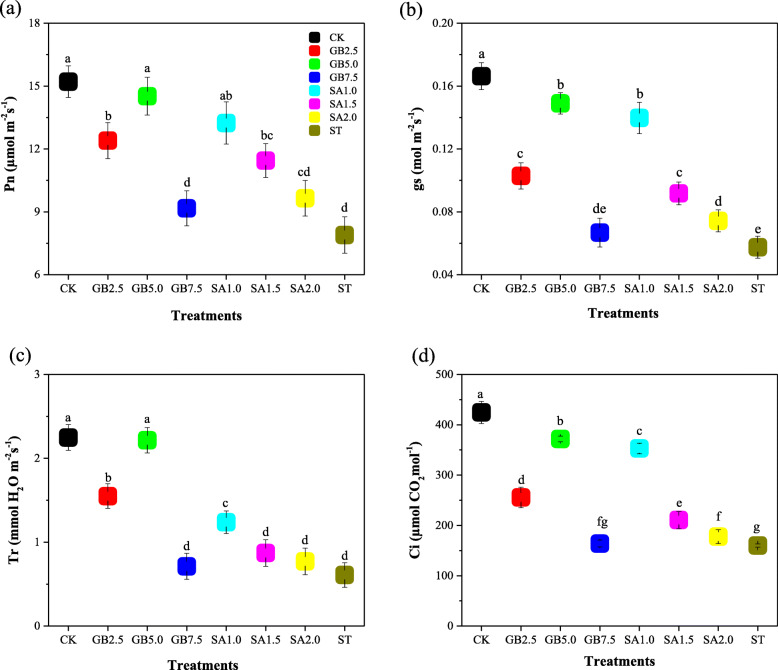
Response of (**a**) net photosynthetic rate (*Pn*), **b** stomatal conductance (*g*_*s*_), **c** transpiration rate (*Tr*) and **d** intracellular CO_2_ concentration (*Ci*) to exogenous glycine betaine (GB) and salicylic acid (SA) under 150 mM NaCl regime. CK = Control; GB2.5 = 2.5 mM GB; GB5.0 = 5.0 mM GB; GB7.5 = 7.5 mM GB; SA1.0 = 1.0 mM SA; SA1.5 = 1.5 mM SA; SA2.0 = 2.0 mM SA; and ST = saline treatment (150 mM NaCl). All data are mean ± standard deviation. Differences between treatments having different letters above the error bars are significant at *P* < 0.05

Net photosynthetic rate (*P*_*n*_), stomatal conductance (*g*_*s*_), transpiration rate (*T*_*r*_), and intracellular CO_2_ concentration (*Ci*) all decreased significantly, by 46, 68, 75, and 62% respectively, in salt stress treatment ST in comparison with CK (Fig. 1). Foliar application of exogenous GB and exogenous SA significantly affected leaf gas exchange parameters under salt stress. Exogenous GB treatments GB2.5 and GB5.0 and exogenous SA treatment SA1.0 significantly increased the values of all cotton leaf gas exchange parameters under salt stress. The highest values of leaf gas exchange parameters were obtained for treatment GB5.0 (Fig. 1).

### Response of photosynthetic enzymes MDA and LRWC to exogenous GB and exogenous SA

**Fig. 2:**
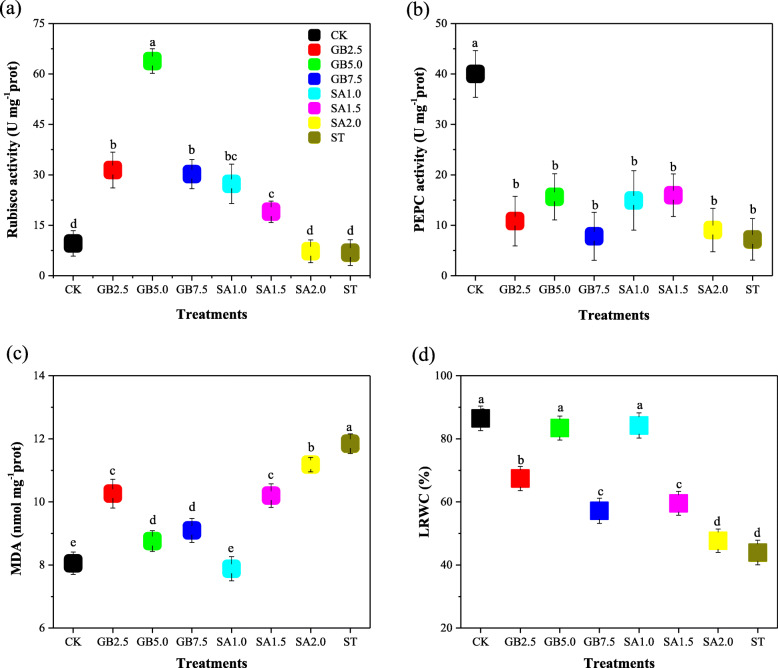
Response of (**a**) D-ribulose-1,5-bisphosphate carboxylase/oxygenase (Rubisco), **b** phosphoenolpyruvate carboxylase (PEPC), **c** malondialdehyde (MDA), and **d** leaf relative water content (LRWC) to exogenous glycine betaine (GB) and exogenous salicylic acid (SA) treatments under salt stress. CK = Control; GB2.5 = 2.5 mM GB; GB5.0 = 5.0 mM GB; GB7.5 = 7.5 mM GB; SA1.0 = 1.0 mM SA; SA1.5 = 1.5 mM SA; SA2.0 = 2.0 mM SA; and ST = saline treatment (150 mM NaCl). All data are mean ± standard deviation. Differences between treatments having different letters above the error bars are significant at *P* < 0.05

Salt stress did not significantly affect D-ribulose-1,5-bisphosphate carboxylase/oxygenase (Rubisco) activity (Fig. 2a). It significantly decreased phosphoenolpyruvate carboxylase (PEPC) and leaf relative water content (LRWC) of cotton seedling leaves by 82 and 49% (Fig. 2b, d) and increased malondialdehyde (MDA) content by 33% in comparison with the well-watered control treatment CK (Fig. 2c). Foliar application of exogenous GB and exogenous SA significantly increased Rubisco activity and decreased leaf MDA content under salt stress. Foliar application of exogenous GB and exogenous SA had no significant effects on PEPC activity but significantly increased LRWC in comparison with treatment ST. After the exogenous GB and exogenous SA treatments, MDA activity remained higher (11.84 nmol/mg prot) for treatment ST. Treatments GB5.0 and SA1.0 were more effective in increasing both Rubisco activity and LRWC and decreasing MDA content.

### Response of chlorophyll fluorescence to exogenous GB and SA

**Fig. 3:**
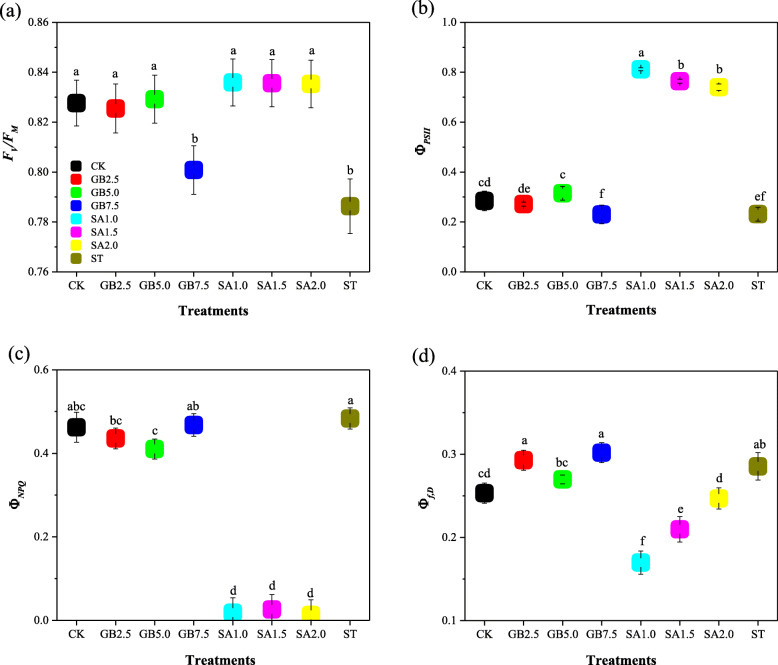
Response to exogenous glycine betaine (GB) and salicylic acid (SA) treatments under salt stress of (**a**) maximal photochemical efficiency of photosystem II (*F*_*V*_*/F*_*M*_), **b** quantum efficiency of photochemical transports used for photosynthesis (Φ_*PSII*_), **c** quantum efficiency of thermal dissipation promoted by the photoprotective non-photochemical quenching via the xanthophyll cycle (Φ_*NPQ*_), and **d** combined quantum efficiency of fluorescence and constitutive thermal dissipation (Φ_*f,D*_). CK = Control; GB2.5 = 2.5 mM GB; GB5.0 = 5.0 mM GB; GB7.5 = 7.5 mM GB; SA1.0 = 1.0 mM SA; SA1.5 = 1.5 mM SA; SA2.0 = 2.0 mM SA; and ST = saline treatment (150 mM NaCl). All data are mean ± standard deviation. Differences between treatments having different letters above the error bars are significant at *P* < 0.05

Chlorophyll fluorescence parameters, including maximal photochemical efficiency of photosystem II (*F*_*V*_/*F*_*M*_), quantum efficiency of photochemical transports used for photosynthesis (Φ_*PSII*_), and combined quantum efficiency of fluorescence and constitutive thermal dissipation (Φ_*f,D*_), were sensitive to treatment ST (salt stress). In contrast, quantum efficiency of thermal dissipation promoted by photoprotective non-photochemical quenching via the xanthophyll cycle (Φ_*NPQ*_) was not significantly affected by salinity. In comparison to CK, both *F*_*V*_/*F*_*M*_ and Φ_*PSII*_ decreased significantly by 5 and 19% in treatment ST, whereas Φ_*f,D*_ greatly increased in treatment ST (Fig. 3). All SA treatments significantly increased both *F*_*V*_/*F*_*M*_ and Φ_*PSII*_ under salt stress. Treatments GB2.5 and GB5.0 significantly increased *F*_*V*_/*F*_*M*_, while only treatment GB5.0 significantly increased Φ_*PSII*_ under salt stress. The parameter Φ_*f,D*_ decreased considerably in treatments SA1.0 and SA1.5 under salt stress. Treatment SA1.0 increased chlorophyll fluorescence the most under salt stress.

### Responses of photosynthetic pigments to exogenous GB and SA

**Fig. 4:**
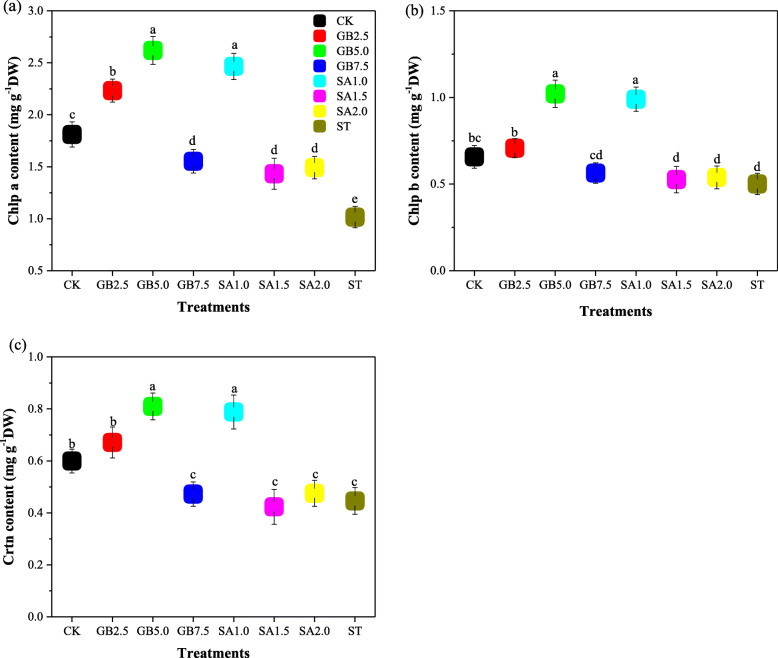
Response to exogenous glycine betaine (GB) and salicylic acid (SA) treatments under salt stress of (**a**) chlorophyll a (chlp a), **b** chlorophyll b (chlp b), and **c** carotenoid (crtn) content. CK = Control; GB2.5 = 2.5 mM GB; GB5.0 = 5.0 mM GB; GB7.5 = 7.5 mM GB; SA1.0 = 1.0 mM SA; SA1.5 = 1.5 mM SA; SA2.0 = 2.0 mM SA; and ST = saline treatment (150 mM NaCl). All data are mean ± standard deviation. Differences between treatments having different letters above the error bars are significant at *P* < 0.05

Variations in chlorophyll a, chlorophyll b (chlp a, chlp b) and carotenoid (crtn) content are shown in Fig. 4. Treatment ST (salt stress only) significantly decreased chlp a, chlp b, and crtn content by 44, 24, and 26% compared to treatment CK. The parameter chlp a, significantly increased with all the concentrations of both foliar applied GB and SA under salt stress. The parameters, chlp b and crtn showed the same trend: they all increased significantly with the treatments GB2.5, GB5.0, and SA1.0 under salt stress. The greatest values of chlp a, chlp b and crtn were given by treatments GB5.0 and SA1.0 (Fig. 4).

### Responses of antioxidant enzymes to exogenous GB and SA

**Fig. 5:**
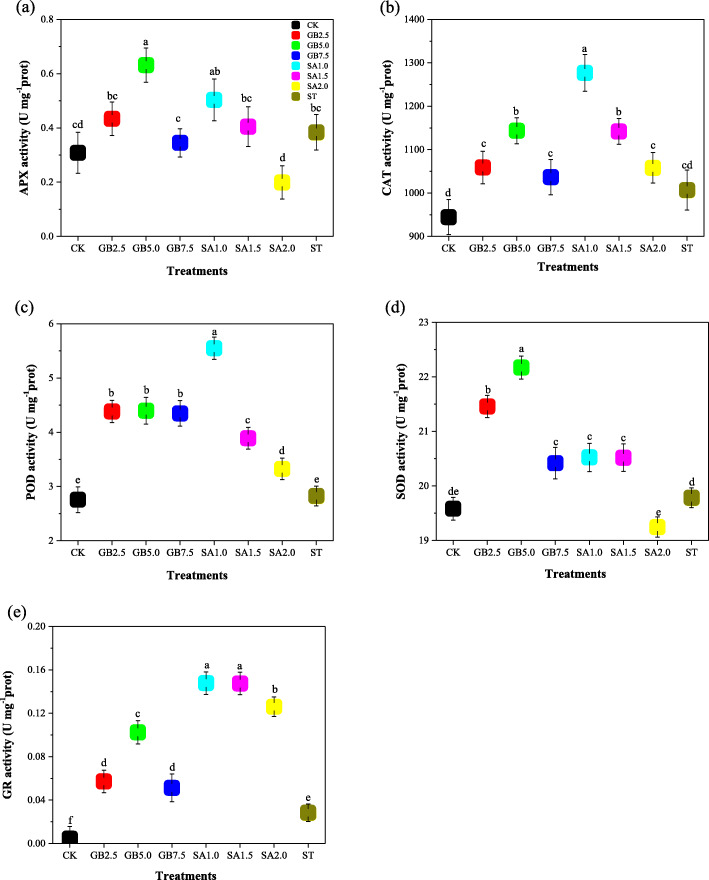
Response of (**a**) ascorbate peroxidase (APX), **b** catalase (CAT), **c** peroxidase (POD), **d** superoxide dismutase (SOD) and **e** glutathione reductase (GR) to exogenous glycine betaine (GB) and salicylic acid (SA) treatments under salt stress. CK = Control; GB2.5 = 2.5 mM GB; GB5.0 = 5.0 mM GB; GB7.5 = 7.5 mM GB; SA1.0 = 1.0 mM SA; SA1.5 = 1.5 mM SA; SA2.0 = 2.0 mM SA; and ST = saline treatment (150 mM NaCl). All data are mean ± standard deviation. Differences between treatments having different letters above the error bars are significant at *P* < 0.05

Antioxidant enzyme activity for the enzymes ascorbate peroxidase (APX), catalase (CAT), peroxidase (POD), superoxide dismutase (SOD), and glutathione reductase (GR) is shown in Fig. 5. APX activity in the cotton leaves was measured after they were harvested; it was not significantly affected by treatment ST, in comparison to CK. Only treatment GB5.0 significantly increased APX activity (Fig. 5a). CAT activity was not significantly affected by treatment ST. However, treatments GB5.0, SA1.0 and SA1.5 significantly increased CAT activity (Fig. 5b). POD activity was not significantly affected by treatment ST but it significantly increased for all treatments under salt stress (Fig. 5c). Similarly, SOD activity was not significantly affected by treatment ST, but of all GB and SA treatments, only SA1.5 did not significantly increase SOD activity (Fig. 5d). GR activity was similar to that of POD in response to GB and SA treatments under salt stress (Fig. 5e).

### Relationships between *P*_*n*_, *F*_*V*_/*F*_*M*_ and chlorophyll a and chlorophyll b content

Under treatment ST, *P*_*n*_*, F*_*V*_/*F*_*M*_, chlp a and chlp b decreased significantly, being reduced by 48, 5, 44 and 24% respectively, in comparison with CK. All four parameters increased as a result of GB and SA treatments under salt stress. Pearson correlations are shown in Table 2. *P*_*n*_ and *F*_*V*_/*F*_*M*_ were significantly positively correlated with chlp a and chlp b (Table 2). On the other hand, a non-significant relationship was observed between *P*_*n*_ and *F*_*V*_*/F*_*M*_.

**Table 2:** Correlations between *Pn* and *F*_*V*_*/F*_*M*_ and chlorophyll a and chlorophyll b

	*Pn*	*F*_*V*_*/F*_*M*_	Chlp a	Chlp b
*Pn*	1	ns	.75**	.72**
*F*_*V*_*/F*_*M*_		1	.45*	.44*
Chlp a			1	.98***
Chlp b				1

*Pn* Net photosynthetic rate, *F*_*V*_*/F*_*M*_ Maximal photochemical efficiency of photosystem II, *Chlp a* Chlorophyll a, *Chlp b* Chlorophyll b

The asterisks *, ** and *** represent significance levels *P* < .05, *P* < .01 and *P* < .001 respectively and *ns* = non-significant

## Discussion

Regulation of leaf gas exchange parameters is an essential aspect of increasing crop resistance to various biotic and abiotic stress conditions [8]. Our analysis of the experimental treatments showed that treatment ST, 150 mM salt stress, caused a significant decrease in cotton leaf gas exchange, while treatments GB5.0 and SA1.0 increased the values of cotton leaf gas parameters under salt stress in comparison with treatment ST (Fig. 1). The salt-induced decreases in leaf gas exchange parameters, including *C*_*i*_*, T*_*r*_*, g*_*s*_*,* and *P*_*n*_, have been observed in *Brassica juncea* L. [25] and *Vigna radiata* L. [26]. These observations are consistent with our results (Fig. 1). Previous investigations of leaf photosynthesis under salt stress showed that foliar application of 0.5 mM exogenous SA protected plant leaf photosynthesis from salt-induced effects [27]. We found that, of the three SA treatments, SA1.0 was most effective in increasing leaf photosynthesis under salt stress. Our results are consistent with those of Nazar et al. [28], who found that a foliar spray of 1.0 mM SA concentration mitigated the salt-induced reduction in leaf photosynthesis for two mung bean cultivars. In contrast, increases in leaf gas exchange parameters, including *g*_*s*_*, P*_*n*_ and *C*_*i*_, from foliar sprayed exogenous GB, have been reported for plants under salt stress [16]. Our results showed that under salt stress, foliar GB application, especially for treatments GB2.5 and GB5.0, significantly increased leaf gas exchange parameter values, which we attribute to increased turgor pressure in cotton leaf guard cells, which increases leaf photosynthesis.

Chlorophyll fluorescence is one of the few physiological parameters that have been shown to correlate with salinity tolerance [29]. Assessing the integrity of photosynthetic apparatus based on chlorophyll fluorescence gives a quick and accurate method of detecting and quantifying plant resistance to environmental stresses [13, 14]. Chlorophyll fluorescence could be used as a nondestructive and noninvasive tool to determine effects of salt stress on the photosynthetic machinery [30]. We found that the 150 mM NaCl salt stress regime significantly influenced the chlorophyll fluorescence parameters of the cotton plants in comparison with the control. The most sensitive index of physiological stress in chlorophyll fluorescence is *F*_*V*_/*F*_*M*_. In treatment ST, *F*_*V*_/*F*_*M*_ decreased significantly from 0.828 to 0.76 when compared with CK. Similar results have been obtained for radishes [31], tomatoes [32], and wheat [33]. We attribute the decrease in *F*_*V*_/*F*_*M*_ to < 0.80 to the light-induced decrease in *P*_*n*_ (photoinhibition) as a response to salt stress. All the SA treatments significantly increased *F*_*V*_/*F*_*M*_ under salt stress when compared with CK, but only treatments GB2.5 and GB5.0 significantly increased *F*_*V*_/*F*_*M*_ under salt stress, to match the increase given by CK. It has been found that foliar sprayed exogenous GB increased *F*_*V*_/*F*_*M*_ in eggplant under salt stress [34] and that foliar sprayed exogenous SA increased *F*_*V*_/*F*_*M*_ in *B. juncea* under salt stress [35]. There are other commonly used fluorescence parameters in addition to *F*_*V*_/*F*_*M*_, including Φ_*PSII*_, Φ_*NPQ*_ and Φ_*f,D*_. We found that Φ_*f,D*_ increased significantly and that Φ_PSII_ decreased significantly under salt stress, but that salt stress had no significant effect on Φ_*NPQ*_. Meggio and Pitacco [36] similarly found that salt stress adversely affected energy partitioning in two genotypes of grapevine rootstock. More investigations are necessary to further analyze energy partitioning in response to foliar spraying of exogenous GB and exogenous SA in salt-stressed plants.

A previous study reported that salinity decreases chlorophyll content by damaging biosynthesis pathways [35]. We observed significant reductions in both chlorophyll a and b content. This observation is consistent with the findings of Zhao et al. [37], who found a decrease in chlorophyll content under salinity. It has been suggested that the decrease in chlorophyll content in salt-stressed plants is due to increased activity of the enzyme chlorophyllase [38]. We found that leaf crtn content decreased significantly in treatment ST. We also found that leaf content of photosynthetic pigments, chlp a, chlp b and crtn, increased for GB and SA treatments; treatments GB5.0 and SA1.0 gave the greatest increases in photosynthetic pigments under salt stress. In a study that produced results consistent with ours, Shaki et al. [39] found that exogenous SA spraying significantly increased chlorophyll content in safflower plants subject to 100 and 200 mM NaCl salt stress regimes. Eraslan et al. [40] demonstrated that foliar spray of 0.5 mM exogenous SA increased crtn content in carrots subjected to NaCl. Contrary to our results, it has been found that foliar spray of GB did not significantly affect chlorophyll a and b content in canola under salinity [41]. This result may have been due to the concentration of exogenous GB and the method of application (the volume sprayed on the leaves of an individual plant).

Quantities of antioxidant enzymes such as POD and SOD, and CAT activity, increased in response to salt stress in order to control salt-induced damage [42, 43]. Elevation of antioxidant enzymes, including SOD, POD and CAT has been reported in many salt-tolerant plant species, such as *Solanum lycopersicum* [44], *Calendula officinalis* [45], and *Jatropha curcas* [46], suggesting a potential function of antioxidants in mitigating salt-induced oxidative damage in plant cells. We found that APX, CAT, SOD, and POD activity slightly increased in response to treatment ST when compared to CK. SOD activity was also found to increase in tomatoes in the presence of salt [47], which is consistent with our findings. In contrast, Hoque et al. [48] observed a decrease in antioxidant enzyme (SOD, POD and CAT) activity under salt stress. We found that the concentration (5.0 mM) of GB and the concentration (1.0 mM) of SA in treatments GB5.0 and SA1.0 significantly increased the activity of APX, CAT, SOD, POD and GR in cotton seedling leaves during salt stress. Khalifa et al. [49] found that spraying with exogenous GB and exogenous SA increased the quantities of antioxidant enzymes (SOD and POD) in lettuce plants subjected to salt stress. Similar results were observed in previous research [20, 48, 50]. It has also been demonstrated that different concentrations of exogenous SA increased the activity of antioxidant enzymes SOD, POD, and CAT in *Nitraria tangutorum* Bobrov subjected to various NaCl regimens [51]. MDA is another primary indicator of oxidative stress, which is usually attributed to oxidative damages [52]. MDA concentrations have been found to increase in maize [53] and barley [54] in response to salinity. We obtained a similar result: MDA content increased significantly in cotton leaves under salt stress. We attribute the cause of the increase in MAD in response to salt stress to the foliar spraying of exogenous GB and exogenous SA inducing additional antioxidant enzyme activity. When sprayed onto leaves under salt stress, treatments GB5.0 and SA1.0 decreased MDA content to a level close to that observed in the control treatment (Fig. 2). Similar concentrations of exogenous SA (0.5 and 1.5 mM) were found to greatly decrease MDA concentration in N. tangutorum plants subjected to salt stress [51]. In contrast, it has also been found that exogenous GB decreased MDA content under salt stress [49].

Under stress, many plant species synthesize considerable amounts of GB and SA to control or repair stress-induced damage. We observed significant accumulations of GB and SA in cotton leaves under salt stress treatment ST. Chen and Murata [55] similarly reported endogenous production of GB in plants to combat environmental stressors. It is known that plants also synthesize SA to mitigate environmental stresses such as salinity [56]. We found that natural accumulations of GB and SA in cotton leaves were insufficient to alleviate the NaCl-induced damage. Our results showed that all GB and SA treatments, except for SA2.0, significantly increased the content of endogenous GB under salt stress. In contrast, endogenous SA content only increased during salt stress when SA treatments were administered to the cotton plants. Khan et al. [57] reported very similar results, that foliar sprayed exogenous SA increased endogenous GB biosynthesis in mung beans (*V. radiata*3) subjected to salt stress. Rice seedlings have also been found to accumulate endogenous GB in their leaves from application of exogenous GB under salt stress [58].

LRWC is an essential indicator of plant physiological water status under salt stress [59]. We investigated the effect of salt stress (treatment ST, 150 mM NaCl) on LRWC of cotton plants to assess the severity of the salt stress treatment on cotton seedlings. Results showed that the ST treatment decreased LRWC (Fig. 2d). Similar results have been obtained for sugar beet grown under 250 mM salt stress [60]. The decrease in LRWC can result in a high accumulation of salt in the rhizosphere, which impedes water uptake by the roots [61]. Studies by Yildirim et al. [62, 63] and Sayyari et al. [64] found that spraying with exogenous GB and exogenous SA increased LRWC in lettuce subjected to water stress and salt stress, results which are consistent with the data shown in Fig. 2d.

## Conclusions

We investigated the effects of foliar spraying of cotton seedlings under salt stress with exogenous GB and exogenous SA on the physiological and biochemical characteristics of cotton seedlings. Of the different concentrations of exogenous GB and SA, the medium concentration (5.0 mM, treatment GB5.0) of GB and the lowest concentration (1.0 mM, treatment SA1.0) of SA showed the greatest increases in leaf gas exchange, pigments content, chlorophyll fluorescence, and antioxidant enzyme activity in cotton plants subjected to salt stress. Foliar spraying of exogenous SA in all treatments significantly increased both endogenous GB content and endogenous SA content during salt stress. In contrast, foliar spraying of exogenous GB increased only endogenous GB content. Therefore, foliar spraying of exogenous SA at a concentration of 1.0 mM is better than foliar spraying of exogenous GB at a concentration of 5.0 mM to alleviate NaCl-induced damage in cotton seedlings because SA better stimulates the accumulation of endogenous GB and SA that provide the plant with important protection against various stress conditions. Based on the outputs of this study, further studies could investigate the effects of 5.0 mM GB and 1.0 mM SA on cotton seedlings subjected to different NaCl regimes. Moreover, to investigate the effects of exogenously applied GB and SA in salt-stressed cotton seedlings growth under natural environmental conditions.

## Methods

### Study site and experimental design

A potted plant experiment was conducted in a phytotron at the Experimental Station of the Farmland Irrigation Research Institute, Chinese Academy of Agricultural Sciences (CAAS) (35°08′N, 113°45′E, altitude 80.77 m), located at Xinxiang City, Henan Province, China. Night and day air temperatures in the phytotron were set to 20 °C and 30 °C, and relative humidity was in the range 50–60%. Photon flux density, calibrated at 350 μmol m^− 2^ s^− 1^ between 06:00 h and 20:00 h, was supplied by LED lamps. The cotton plants were *Gossypium hirsutum* L. var. Xinlunzhong-37, a leading variety in the salty soil area of Southern Xinjiang. Seeds were purchased from Tahe (Seed CO., LTD), Alaer city in Xinjiang province. The cotton seeds were disinfected in 0.3% hydrogen peroxide for 30 min and then rinsed three times with deionized water. Seeds were sown in a nursery for germination, and 6–7 day old uniform seedlings were transplanted to pots at one plant per pot. The plastic pots used, 16 cm diameter and 18 cm high, were each filled with ~ 2.5 kg dry sandy soil.

No treatment was administered to the plants during the early growing stage, before 20 days after transplanting (DAT). Each pot was irrigated with 150 mL of half-strength Hoagland solution every 2–3 days. The composition of the nutrient solution was: 236.2 g L^− 1^ Ca(NO_3_)_2_4H_2_O, 101.1 g L^− 1^ KNO_3_, 40 g L^− 1^ NH_4_NO_3_, 61.6 g L^− 1^ MgSO_4_7H_2_O, 34 g L^− 1^ KH_2_PO_4_, 18.6 g L^− 1^ KCl, 3.671 g L^− 1^ Fe-EDTA and various microelements (1.546 g L^− 1^ H_3_BO_3_, 0.396 g L^− 1^ MnCl_2_4H_2_O, 0.575 g L^− 1^ ZnSO_4_7H_2_O, 0.125 g L^− 1^ CuSO_4_5H_2_O, 0.036 g L^− 1^ CoCl_2_6H_2_O, and 0.093 g L^− 1^ (NH4)6M_O7_O_24_4H_2_O) [65–67]. The experimental design consisted of two salt concentrations (0 and 150 mM), three concentrations of GB (2.5, 5.0 and 7.5 mM), and three concentrations of SA (1.0, 1.5, and 2.0 mM), with the control of a well-watered treatment with zero salt concentration (CK). The experiment was conducted as a completely randomized design with three replicates. The treatment labels were: CK = Control; GB2.5 = 2.5 mM GB; GB5.0 = 5.0 mM GB; GB7.5 = 7.5 mM GB; SA1.0 = 1.0 mM SA; SA1.5 = 1.5 mM SA; SA2.0 = 2.0 mM SA; and ST = saline treatment (150 mM NaCl). During 20, 22, and 25 DAT, to avoid the effects of a sudden impact of a 150 mM NaCl concentration, the plants were irrigated to 90% field capacity with 50, 100, and 150 mM NaCl in Hoagland solution. From the first day of ST to harvest, the salt concentration was maintained at a constant level (150 mM) of salt stress. During 10 days after ST application, liquid solutions of GB at the three different concentrations and SA at the three different concentrations were sprayed daily onto the leaves at 5.0 mL per plant according to the experimental treatments. Within the 10-day period of exogenous GB and SA foliar treatments under the 150 NaCl salt stress regime, leaf gas exchange and chlorophyll fluorescence parameters were measured four times. Plants were harvested for determination of biochemical parameters after initialization to the 150 mM NaCl regime (salt stress) and being sprayed with exogenous GB and SA for 10 days.

### Measurements

#### Leaf gas exchange and chlorophyll fluorescence

The parameters *P*_*n*_*, g*_*s*_*, T*_*r*_ and *C*_*i*_ of the third fully expanded leaves of the cotton plants were measured at the same time, between 09:00 h and 11:00 h, with a Li-6400XT portable photosynthesis system (Li-COR Inc., Lincoln, NE, USA). The parameters were measured every 3 days during the period from 20 DAT to harvest under a light flux density of 1200 μmol m^− 2^ s^− 1^, block temperature of 25 °C, and airflow of 500 μmol s^− 1^. During the measurement period, tubes containing desiccant and soda were used to control H_2_O and CO_2_ inside the instrument chamber. The measurement used the CO_2_ mixer, it was set to control the reference concentration with a target of 400 μmol mol^− 1^ and the soda lime knob was on full scrub position. The H_2_O sample inside the chamber is the leaf H_2_O and the desiccant lime knob was set at full bypass position. Chlorophyll fluorescence, in the form of leaf gas exchange parameters, was measured every 3 days during the same period using a MINI-PAM-II fluorimeter (photosynthetic yield analyzer). The activity of photosystem II (PSII) was determined by *F*_*V*_*/F*_*M*_, together with minimal chlorophyll fluorescence (F_o_) and maximal chlorophyll fluorescence (F_m_). F_o_ and F_m_ were measured when the leaves had been dark-adapted for 30-min, using the dark leaf clip DLC-8, and F′_m_ was measured under full illuminating light. Quantum efficiency of photochemical transports used for photosynthesis (Φ_*PSII*_), quantum efficiency of thermal dissipation promoted by the photoprotective non-photochemical quenching via the xanthophyll cycle (Φ_*NPQ*_), and combined quantum efficiency of fluorescence and constitutive thermal dissipation (Φ_*f,D*_) were calculated by:

1 $$ {\Phi}_{PSII}=\frac{\left({F}_m^{,}-{F}_s\right)}{F_m^{,}} $$

2 $$ {\Phi}_{NPQ}=\left(\frac{F_s}{F_m^{,}}\right)-\left(\frac{F_s}{F_m}\right) $$

3 $$ {\Phi}_{f,D}=\frac{F_s}{F_m} $$

### Key photosynthetic enzymes, photosynthetic pigments, GB and SA contents

After leaf harvesting, Rubisco activity was measured using the method described by McCurry et al. [68]. PEPC was measured following Stiborová [69]. Determination of chlorophyll a and chlorophyll b (chlp a, chlp b) and carotenoid (crtn) content of cotton leaves followed the methods described in details by Harder et al. [70]. Wiley mill was used to grind the dry leaves to pass a 40 (unit) mesh screen. After receiving a constant weight by oven-drying, the samples were stored over calcium chloride, and weighed to 0.1 ± 0.0003 g. A Beckman DK-2A Spectrophotometer was used to determine the optical densities (O.D.) at 663, 645, and 460 nm. Milligrams of chlp a and chlp b were obtained after solving simultaneous equations designed for the absorption coefficients at 663 and 645 nm, respectively and the crtn content was immediately calculated from the absorption coefficient at 460 nm [70]. GB was converted to its *n*-butyl ester and determined by fast atom bombardment using the method described by Nuccio et al. [71]. The sampling and quantification of leaf endogenous SA used the procedure described by Molina et al. [72].

### Antioxidant enzymes, MDA and LRWC

About 0.5 g of fresh leaf tissue was ground using a pestle and mortar. An amount of 5.0 mL of 0.05 M precooled phosphate buffer (pH 7.8) was added to the homogenate, which was then centrifuged at 15000 g at 4 °C for 20 min. SOD activity was determined spectrophotometrically from the inhibition of the photochemical reduction of nitroblue tetrazolium (NBT) at 560 nm [73]. POD activity was measured by the determination of guaiacol oxidation by H_2_O_2_ at 470 nm [74]. CAT activity was measured by monitoring the disappearance of H_2_O_2_ at 240 nm [75]. To measure APX activity, frozen leaves were homogenized in 5.0 mL of 50 mM Tris-HCl buffer (pH 7.0) with 1.0 mM sodium ascorbate, 1 mM DTT, 1.0 mM EDTA, 1 mM reduced glutathione, 5.0 mM MgCl_2_, and 1% PVPP (w/v) at 4 °C [76]. GR was determined by measuring the glutathione-dependent oxidation of NADPH at 340 nm in a reaction mixture containing 950 μL of 0.15 mM NADPH, 0.5 mM GSSG, and 3 mM MgCI_2_ in 50 mM Tris-HCl (pH 7.5) and 50 μL extract [77]. Lipid peroxidation was measured spectrophotometrically from malondialdehyde (MDA) content using a thiobarbituric acid (TBA) reaction following the method described by Heath and Packer [78].

To determine LRWC, leaf samples (the sixth leaf from the top of the plant) were collected from three plants per treatment. Individual leaves were cut from the stem and immediately weighed to determine FW. To obtain the turgid weight (TW), leaves were floated in deionized water inside a closed petri dish. Water was periodically wiped from the leaf surface using absorbent paper until a steady state weight was observed. Leaf samples were oven-dried at 75 °C for 72 h to determine DW. LRWC was calculated using the following equation (Kaya et al. [79]):

4 $$ \mathrm{LRWC}\ \left(\%\right)=\left[\left(\mathrm{FW}-\mathrm{DW}\right)/\left(\mathrm{TW}-\mathrm{DW}\right)\right]\times 100 $$

### Experimental soil pH and EC

Soil pH and EC were measured by a pH meter (Mettler Toledo 320-S Shanghai Bante Instrument Co., Ltd., China). Soil pH and EC were measured in the initial soil sample and during all the saline treatments (150 mM NaCl) and after harvesting to allow us to assess the variation and final values in the soil after harvesting. Soil pH was alkaline and remained almost constant during the whole experiment (from 8.12 to 8.56). Evaporation and transpiration slightly increased EC accumulation in the soil by 1% above the expected value from the first NaCl application to harvesting (5.42 to 5.46 dS m^− 1^).

### Statistical analysis

One-way analysis of variance (ANOVA) was performed using SPSS 23.0 (IBM Corporation, New York, NY, USA). Correlations between the different treatments were determined by Pearson correlation using SPSS 23.0. All data are expressed as mean (*n* = 3) ± standard deviation, and Duncan’s multiple range test was performed to differentiate the treatments at a 5% significance level.

## Data Availability

The datasets used and/or analysed during the current study are available from the corresponding author on reasonable request.

## Abbreviations

APX: Ascorbate peroxidase
CAT: Catalase
Chlp: Chlorophyll
*Ci*: Intracellular CO_2_ concentration
DW: Dry weight
FW: Fresh weight
F_m_: Maximal fluorescence
F_o_: Minimal fluorescence
F_s_: Steady-state chlorophyll fluorescence
*F*_*V*_*/F*_*M*_: Maximal photochemical efficiency of photosystem
GB: Glycine betaine
GR: Glutathione reductase
g_s_: Stomatal conductance
LRWC: Leaf relative water content
MDA: Malondialdehyde
PEPC: Phosphoenolpyruvate carboxylase
*P*_*n*_: Net photosynthetic rate
POD: Peroxidase
Rubisco: D-ribulose-1,5-bisphosphate carboxylase/oxygenase
SA: Salicylic acid
SOD: Superoxide dismutase
*T*_*r*_: Transpiration rate
Φ_*PSII*_: Quantum efficiency of photochemical transports used for photosynthesis
Φ_*NPQ*_: Quantum efficiency of thermal dissipation promoted by the photoprotective non-photochemical quenching via the xanthophyll cycle
Φ_*f,D*_: Combined quantum efficiency of fluorescence and constitutive thermal dissipation
